# A practical CD90.2-based FACS strategy for obtaining Schwann cell-enriched cultures from adult mouse nerves

**DOI:** 10.3389/fncel.2026.1873287

**Published:** 2026-07-27

**Authors:** Karen Libberecht, Hanne Jeurissen, Nathalie Dirkx, Yara Lambrechts, Lise Van Horebeek, Koen Kuipers, Femke Reubens, Jana Van Broeckhoven, Tim Vangansewinkel, Esther Wolfs

**Affiliations:** 1 Biomedical Research Institute (BIOMED), Hasselt University - UHasselt, Diepenbeek, Belgium; 2 Laboratory of Neurobiology, VIB-KU Leuven Center for Neuroscience, Leuven, Belgium; 3 Department of Neurosciences, Experimental Neurology and Leuven Brain Institute (LBI), KU Leuven - University of Leuven, Leuven, Belgium; 4 Peripheral Neuropathy Research Group, Department of Biomedical Sciences, University of Antwerp, Antwerp, Belgium

**Keywords:** cell culture, flow cytometry, mouse, peripheral nervous system, primary cells, Schwann cell isolation

## Abstract

Primary Schwann cell cultures derived from adult murine peripheral nerves are frequently compromised by fibroblast overgrowth, limiting their utility for *in vitro* studies. Existing enrichment strategies often rely on selective growth conditions, prolonged culture, or antibody-mediated positive selection, which may alter Schwann cell physiology, trigger unwanted signaling, or result in incomplete fibroblast removal. To address these limitations, we developed a reproducible negative selection protocol for the isolation and enrichment of primary adult murine Schwann cells using fluorescence-activated cell sorting (FACS). By specifically targeting contaminating fibroblasts using the marker CD90.2 (Thy-1.2), these cells are efficiently depleted while leaving the Schwann cell population unbound by antibody complexes. This approach enables the rapid generation of highly enriched Schwann cell populations without the need for extended *in vitro* expansion or antimitotic agents. By avoiding direct receptor-ligand interactions on Schwann cells, this method preserves their native physiological state and surface epitopes, supporting sensitive downstream applications. Successful Schwann cell enrichment was confirmed by quantitative PCR, western blot and immunocytochemistry, demonstrating the enrichment of Schwann cell-specific markers and minimal fibroblast contamination. Overall, this approach enables the rapid generation of highly enriched Schwann cell populations while preserving features relevant to mature Schwann cell biology. This protocol provides a robust and practical tool for researchers requiring physiologically representative adult Schwann cells for molecular, functional, and co-culture-based studies of the peripheral nervous system.

## 1 Introduction

Peripheral nerve research and the advancement of regenerative medicine depend on access to high-quality Schwann cell cultures. As the primary glial cells of the peripheral nervous system (PNS), Schwann cells provide essential trophic support to axons, maintain the myelin sheath, and orchestrate the complex multicellular response to nerve injury (Bosch-Queralt et al., 2023). Access to well-defined primary Schwann cell cultures is therefore a prerequisite for research on peripheral neuropathies, nerve regeneration, and emerging cell-based therapies (Morrissey et al., 1991; Hyung et al., 2015; Endo et al., 2022; Negro et al., 2022). However, obtaining primary Schwann cell cultures from adult peripheral nerves remains technically challenging (Monje, 2023). Following enzymatic dissociation and/or mechanical dissociation, the resulting suspensions are heterogeneous. In peripheral nerve-derived cultures, the primary contamination consists of fibroblasts, which are abundant within the connective tissue layers of the epineurium, perineurium, and endoneurium surrounding the peripheral nerve (Monje, 2023; Metafune et al., 2025). These cells pose a significant hurdle due to their superior proliferative rate, allowing them to rapidly outpace and dominate the culture, especially in adult peripheral nerves, where fibroblasts are most abundant. Despite these challenges, the use of Schwann cells isolated from adult tissue is increasingly recognized as crucial for accurately modeling the PNS (Endo et al., 2022; Negro et al., 2022). Previous techniques to limit fibroblast expansion have predominantly relied on conventional, nonspecific methods. These include chemical approaches, such as the use of anti-mitotic or cAMP-elevating compounds, as well as physical protocols, such as the “cold-jet” technique, in which ice-cold culture medium is rapidly added and aspirated to selectively detach less adherent cells. However, these methods frequently subject the culture to extreme mechanical, thermal, and chemical stress. Given that Schwann cells are notoriously sensitive to such environmental fluctuations, these approaches often induce off-target toxicity, compromise cellular viability, and alter the fundamental physiology of the cells. Furthermore, they typically fail to achieve complete fibroblast removal, leaving a residual population that hinders the acquisition of the highly purified populations required for sensitive downstream analysis (Jirsová et al., 1997; Teare et al., 2004; Shen et al., 2012).

In recent years, alternative strategies such as immunopanning, magnetic separation, and flow cytometry have been developed to improve Schwann cell enrichment, largely by targeting positive selection markers, including the Schwann cell marker P75 neurotrophin receptor (P75) (Spiegel and Peles, 2009; Lutz, 2014; Andersen et al., 2016; Tomlinson et al., 2020). While these specialized approaches can achieve high purity, they often require multiple rounds of manipulation or depend on the availability of transgenic reporter mouse lines, limiting their broad applicability. Beyond these technical constraints, a more critical concern is the inherent nature of positive selection, where target cells remain coated with antibodies or magnetic beads, which can physically obstruct subsequent immunostaining, mask essential surface epitopes, and distort multiparametric functional readouts. Additionally, previous research demonstrates that such direct receptor-ligand interactions can alter the cellular state, as cells isolated via negative selection or FACS-based depletion exhibit transcriptional signatures that are significantly more representative of their natural physiology than those obtained through positive selection (Elkord et al., 2005; Beliakova-Bethell et al., 2014). This underscores the importance of avoiding direct cell manipulation during isolation to maintain physiological integrity.

To address these limitations, we identified negative selection via CD90.2 (Thy-1.2) depletion as a superior alternative for Schwann cell purification. CD90.2 is strongly expressed by peripheral nerve fibroblasts but shows little to no expression in Schwann cells (Monje, 2020). Leveraging this differential expression, CD90.2-based negative selection enables the efficient depletion of the fibroblast population without directly targeting Schwann cell surface receptors. Hence, by isolating the CD90.2-negative fraction, the recovered Schwann cells remain entirely unbound to antibodies or magnetic beads. This hands-off approach not only preserves the unperturbed physiological state of the cells but also ensures that surface epitopes remain accessible, thereby facilitating seamless integration into sensitive downstream applications.

In this study, we establish a robust and reproducible CD90.2-based depletion workflow designed to isolate high-purity Schwann cell populations from adult murine peripheral nerves, including both sciatic nerve and brachial plexus. By employing a single-step FACS-based depletion after a minimal recovery period, this method consistently yields enriched populations with exceptional purity across experimental replicates. We validate the cellular identity of the sorted population at both transcriptional and translational levels, confirming their suitability for a broad range of molecular and cellular assays. Ultimately, this protocol provides a high-efficiency resource for laboratories requiring adult Schwann cell cultures with minimal *in vitro* manipulations.

## 2 Materials and equipment

The materials and equipment used for murine peripheral nerve isolation and subsequent cell sorting are listed in Table 1.

**TABLE 1:** A comprehensive list of materials and equipment used for the murine peripheral nerve isolation and subsequent cell sorting.

Product/item	Details and company
Cell culture media and supplements	
Dulbecco’s modified Eagle medium, high glucose (DMEM)	41966-029, Gibco, Thermo Fisher Scientific, Waltham, MA, USA
Leibovitz’s medium (L15)	11415-064, Gibco
Heat-inactivated fetal bovine serum (FBS)	SxxxH, Biowest, Nuaillé, France
Penicillin-streptomycin (P/S)	15140-122, Gibco
Hank’s balanced salt solution (HBSS)	H8264/H9394, Sigma-Aldrich, St. Louis, MO, USA
Phosphate-buffered saline (PBS)	392-0433, VWR International, Radnor, PA, USA
Growth factors	
Basic fibroblast growth factor (bFGF)	11343625, ImmunoTools, Friesoythe, Germany
Neuregulin-1B (NRG)	11343045, ImmunoTools
Recombinant human platelet-derived growth factor-AA (PDGF-AA)	11343685, ImmunoTools
Forskolin	100-0249, Stemcell technologies, Vancouver, Canada
Enzymes and dissociation reagents	
Collagenase type I	SCR103, Sigma-Aldrich
Dispase II	D4693-1G, Sigma-Aldrich
Trypsine-EDTA	25300-062, Gibco
Coating and staining reagents	
Poly-L-lysine solution (PLL)	P4832, Sigma-Aldrich
CD90.2-FITC primary antibody	140303, Biolegend, San Diego, CA, USA
Trypan blue	15250-061, Gibco
General chemicals and anesthetics	
Ethanol 70%	64-17-5, Magis-Pharma, Antwerp, Belgium
Isoflurane	Isoflutek 1,000 mg/g, Laboratorios Karizoo, Barcelona, Spain
Surgical tools	
Dumont #5SF forceps	11252-00, Fine Science Tools, Heidelberg, Germany
Graefe forceps	11050-10, Fine Science Tools
Student Vannas spring scissors	91500-09, Fine Science Tools
Student surgical scissors	91400-10, Fine Science Tools
Labware and consumables	
15 ml tubes	188271-N, Greiner Bio-One, Kremsmünster, Austria
6-well plates	657160, Greiner Bio-One
Falcon^®^ 5 ml tubes	352054/352235, Corning Life Sciences, USA
Fuchs-Rosenthal counting chamber	BR719805, Sigma-Aldrich
Petri dishes	82.1473.001, Sarstedt, Nümbrecht, Germany
Pipettes	83.1172.010, Sarstedt
Polypropylene 5 ml tubes	55.526.006, Sarstedt
Polystyrene 5 ml tubes with cell strainer cap	352235, Falcon, Corning Life Sciences, Durham, NC, USA
Flow cytometry and other equipment	
100 μm nozzle	647341, BD Biosciences, San Jose, CA, USA
BD FACS accudrop beads	345249, BD Biosciences
BD FACSDiva CS&T beads	65505, BD Biosciences
Cell sorter	BD FACSAria Fusion cell sorter, BD Biosciences
Centrifuge	5804 R, Eppendorf, Hamburg, Germany
CO_2_ incubator	MCO-18AIC (UV), Sanyo, Rotselaar
Inverted microscope (imaging)	Nikon Eclipse Ti2-E, Nikon Instruments, Tokyo, Japan
Inverted microscope (cell culture)	ZE-PRIMO-BF-05, primovert, Carl Zeiss Microscopy GmbH, Jena.
Stereomicroscope	Leica S6 E, Leica Microsystems, Wetzlar, Germany
Vertical laminar flow cabinet	FlowFAST V, Faster S.r.l., Cornaredo, Italy

## 3 Methods

### 3.1 Overview of the workflow

**FIGURE 1:**
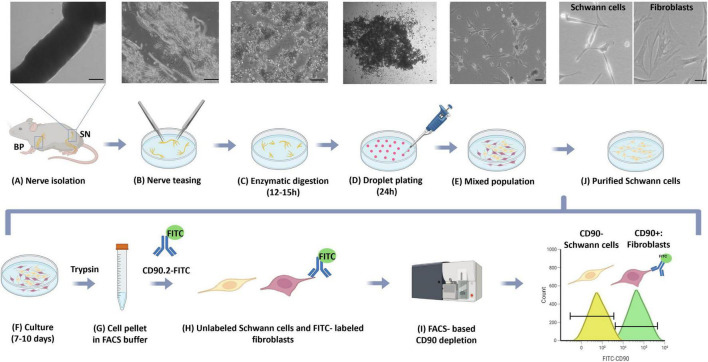
Schematic workflow for the isolation and negative selection-based enrichment of adult murine Schwann cells. Sciatic nerves and brachial plexuses are dissected **(A)**, mechanically teased **(B)**, and enzymatically digested **(C)** to generate a single cell suspension. Primary cells are seeded using a droplet plating approach on PLL-coated surfaces and maintained for 7–10 days to allow cell recovery, growth and myelin clearance prior to sorting **(D–F)**. Subsequently, these heterogeneous cultures are harvested, resuspended in FACS buffer **(G)**, and processed via FACS utilizing CD90.2-FITC (Thy-1.2) as a fibroblast-enriched marker, enabling depletion of CD90.2^+^ cells **(H,I)**. The CD90.2^–^ fraction is collected as the Schwann cell-enriched population and used for downstream analyses **(J)**. Representative phase-contrast images of cultures before and after sorting are shown. Scale bar **(A–F)** = 50 μm. SN, sciatic nerve; BP, brachial plexus. Figure created with BioRender. Jeurissen, H. (2026) (https://BioRender.com/juwkfqz) is licensed under CC BY 4.0.

Figure 1 provides a schematic overview of the workflow used to isolate and enrich primary Schwann cells from adult mouse peripheral nerve tissue. Sciatic nerves and brachial plexuses were dissected and subjected to mechanical nerve teasing followed by enzymatic dissociation to obtain a single cell suspension. To increase cell attachment and survival, cells were plated using a droplet plating approach on PLL-coated surfaces. Moreover, to allow clearance of myelin debris, cells were maintained in short-term culture for 7–10 days (Monje, 2023). Mixed cultures were subsequently processed by FACS using CD90.2 as a fibroblast-enriched marker, enabling negative selection through depletion of CD90.2^+^ cells while leaving the Schwann cell population unbound by antibody complexes. The CD90.2^–^ fraction was collected as the Schwann cell-enriched population. For this study, the pre-sorted mixed population and the CD90.2^+^ cells were additionally collected for comparison and validation.

This workflow allows enrichment of Schwann cells without direct targeting of Schwann cell surface markers, thereby minimizing potential alterations in cellular state and preserving surface epitopes for downstream applications. Recovered cells were used either directly for molecular analyses or reseeded on coated surfaces for further characterization.

### 3.2 Animals

Experiments were performed using both male and female adult C57BL/6J mice, ranging from 8 to 12 weeks of age. Animals were housed under standard conditions with free access to food and water. All experimental animal procedures were approved by the Ethical Committee for Animal Experimentation (ECAE) of Hasselt University (reference number 202046KA1) and were performed in accordance with the guidelines set out in Directive 2010/63/EU on the protection of animals used for scientific purposes.

### 3.3 Step-by-step procedures

Notes:

- To avoid cell contamination, the entire procedure needs to be performed in sterile conditions.
- The number of animals needed will depend on the downstream application. For immunohistochemistry and qPCR it is recommended to pool minimally 2 to maximum 5 mice. For western blot, it is recommended to pool material from minimally 5 ranging to 10 mice. These estimates are based on the collection of both sciatic nerves and brachial plexus from each animal.

(A) Day before isolation

Coating of culture ware

- Prepare the required number of PLL-coated petri dishes based on a ratio of 1 dish per 2 mice.
- Dilute the PLL stock solution 1:10 in sterile PBS.
- Add approximately 8 ml of diluted PLL to each petri dish and incubate at room temperature (RT) for at least 30 min.
- Aspirate the PLL solution and wash the dishes three times with sterile MilliQ (MQ).
- Allow the dishes to dry overnight under sterile conditions at RT.

Reagent preparation

- Prepare the enzymatic digestion stock solution (10×) by dissolving 250 mg dispase II and 50 mg collagenase type I in 10 ml DMEM, without FBS. Sterile-filter the solution through a 0.22 μm filter. Aliquot and store at −20 °C until use.
- Prepare standard medium consisting of DMEM supplemented with 10% FBS, 100 U/ml penicillin, and 100 μg/ml streptomycin.
- Prepare Schwann cell medium by supplementing standard medium with 2 μM forskolin, 10 ng/ml NRG, and 5 ng/ml PDGF-AA.
- Prepare HBSS/FBS neutralization medium containing 40% HBSS and 60% FBS.

Dissection equipment

- Sterilize all dissection instruments before use, including:
  ○ Student standard surgical scissors
  ○ Student Vannas spring scissors
  ○ Graefe forceps
  ○ Dumont #5SF super fine tip forceps

(B) Day of isolation

Preparation

1. Wipe all working surfaces and equipment with 70% ethanol.
2. Prepare all required media in advance (see preparation or reagents) and keep L15 on ice.
3. Thaw the 10× enzymatic stock solution on ice (see reagent preparation in A).
4. Prepare a 6-well plate containing 2 ml ice-cold L15 medium per well (1 plate per 2–4 mice).
5. Keep the plate on ice throughout the isolation process to prevent a reduction in cell viability.

Collection of tissues

1. Sacrifice mice by cervical dislocation or another approved euthanasia method in accordance with institutional ethical guidelines.
2. Spray the fur with 70% ethanol and place the mouse in a laminar flow cabinet.
3. Position the mouse under a dissection microscope:
- Prone position for sciatic nerve isolation.
- Supine position for brachial plexus isolation.
6. Secure the hindlimbs and forelimbs in an extended position (pinned or taped) to facilitate exposure for isolation.

Sciatic nerve isolation

1. Make a small incision along the posterior thigh using surgical scissors.
2. Gently separate surrounding muscle layers using blunt dissection tools.
3. Identify the sciatic nerve at the sciatic notch and trace it distally to its trifurcation.
4. Carefully free the nerve from surrounding connective tissue, muscle, fat, and blood vessels using fine forceps and Vannas spring scissors, avoiding mechanical damage.
5. Transect the nerve proximally, then gently elevate it to facilitate controlled dissection along its course toward the distal end.
6. Complete the procedure by transecting the distal end of the nerve.
7. Immediately transfer the isolated nerves into the 6-well plate containing ice-cold L15 medium using Dumont #5SF forceps.

Brachial plexus isolation

1. Position the mouse supine and open the thoracic inlet.
2. Expose the axillary region using standard surgical scissors, and carefully remove overlying connective tissue using Vannas spring scissors.
3. Identify the brachial plexus branches between the clavicle and the first rib.
4. Isolate the nerve in a proximal-to-distal direction, freeing it from surrounding connective tissue, muscle, fat, and blood vessels using fine forceps and Vannas spring scissors. Avoid mechanical damage.
5. Transect the nerve proximally, then gently elevate it to facilitate controlled dissection along its course toward the distal end.
6. Complete the procedure by transecting the nerve distally.
7. Immediately transfer the isolated nerve into a 6-well plate containing ice-cold L15 medium using a Dumont #5SF forceps.

Nerve trimming and removal of connective tissue

1. Place the isolated nerves under a stereomicroscope.
2. Remove visible epineurial fat and connective tissue using Dumont #5SF super fine tip forceps.
3. Remove the epineurium as a single intact sheath, avoiding stretching or compression of the nerve to prevent damage to the nerve fibers, using Dumont #5SF super fine tip forceps.
4. Transfer the cleaned nerves into a new well containing fresh L15 medium on ice to rinse the tissue.

Mechanical teasing of the nerve fibers

1. Dilute the 10× enzymatic cocktail to a 1× concentration in DMEM without FBS.
2. Transfer the nerves into a 6-well plate containing 2 ml/well of 1× enzymatic cocktail in DMEM (see reagent preparations).
3. Tease the nerve fibers by pulling away individual fascicles using both Dumont #5SF super fine tip forceps and Graefe forceps.
4. Continue teasing until the fascicles are separated into individual nerve fibers, regardless of their original diameter, as demonstrated in Figure 1B.

Enzymatic digestion

1. Incubate the teased nerve fibers in the enzymatic digestion mix at 37 °C and 8.5% CO_2_ for 12–15 h. Determine the exact incubation time by the visual appearance of the cells, aiming for a degree of dissociation consistent with Figure 1C.
2. After digestion, transfer the digested nerves and medium into a 15 ml tube.
3. Terminate enzymatic activity by adding HBSS/FBS (see reagent preparations), using a total volume equal to twice the digestion volume (1:2 ratio).
4. Transfer the rinsing solution to the 15 ml tube containing the digested tissue.
5. Centrifuge the cell suspension at 200×*g* at 4 °C for 10 min.
6. Discard the supernatant and resuspend the pellet in 5 ml standard medium (see reagent preparations).
7. Centrifuge again at 200×*g* at 4 °C for 10 min.
8. Finally, resuspend the pellet in Schwann cell medium (see reagent preparations) in a volume of 750 μl per mouse.

Droplet plating and short-term recovery culture

1. To increase Schwann cell survival and attachment, plate the cell suspension as evenly distributed 20–30 μl droplets onto PLL-coated petri dishes.
2. Carefully place the droplets without disrupting their structure and incubate the dishes at 37 °C and 8.5% CO_2_ (Andersen and Monje, 2018).
3. On the following day, gently add 10 ml of Schwann cell medium to each petri dish.
4. Maintain the culture for 7–10 days at 37 °C and 8.5% CO_2_ prior to sorting (Andersen and Monje, 2018). During this period, monitor the removal of debris and cell growth, the culture should progress from a debris-rich appearance, as shown in Figure 1D, to a confluent mixed population suitable for sorting, as illustrated in Figure 1E.
5. Replace the Schwann cell medium three times per week, without adding antimitotic agents. Due to routine media changes, the myelin debris will be gradually removed over time, allowing cell growth and expansion. Minimize the time dishes remain outside the incubator, as Schwann cells are sensitive to pH changes caused by CO_2_ loss (indicated by medium color changes). During the first week of culture, debris should gradually decrease, and cells typically adopt a spindle-shaped, bipolar morphology.

(C) Day before FACS sorting

Reagent preparation

- Prepare sterile FACS buffer consisting of ice-cold PBS supplemented with 2% FBS. Keep on ice until use.
- Prepare the required volume of Schwann cell medium for downstream culture and standard medium supplemented with 10% FBS (20% FBS final concentration) for coating the polypropylene tubes.

Coating and culture plates

- To maximize cell recovery and prevent adhesion to the tube walls, pre-coat polypropylene FACS collection tubes with Schwann cell medium supplemented with an additional 10% FBS (20% FBS final concentration).
- Incubate the tubes for at least 1 h at RT.
- Immediately before sorting, aspirate two-thirds of the coating medium while ensuring that the interior surfaces remain moist.

Preparation for downstream assays

- If cells are intended for continued culture (e.g., imaging or proliferation assays), prepare PLL-coated glass coverslips or T25 flasks in advance.
- If cells are intended for direct downstream analyses [e.g., quantitative polymerase chain reaction (qPCR) or western blot], no coating is required, and cells can be collected as a pellet or directly into lysis buffer.

(D) FACS-based enrichment of Schwann cells by CD90.2 depletion

**FIGURE 2:**
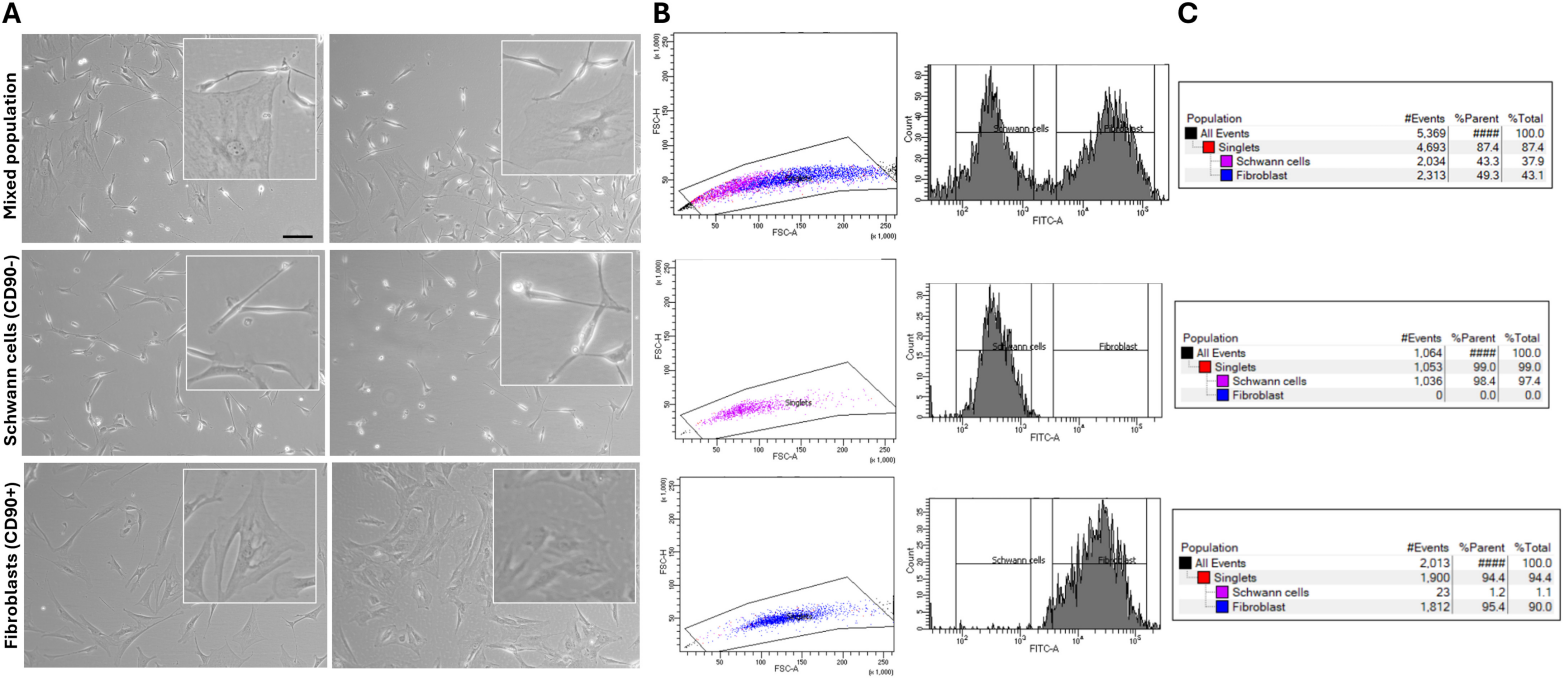
Cell morphology and purity before and after CD90.2-based sorting. **(A)** Representative phase-contrast images of mixed cultures prior to sorting (upper panel), the CD90.2^–^ Schwann cell-enriched fraction (middle panel), and the CD90.2^+^ fibroblast-enriched fraction (bottom panel). Insets show higher magnifications of individual cells. Scale bar = 100 μm. **(B)** Flow cytometry gating of the corresponding populations. The gating strategy involved the exclusion of debris and cell aggregates via forward and side scatter (FSC-A vs. SSC-A), followed by doublet exclusion using singlet discrimination (FSC-H vs. FSC-A). Pre-sorted cultures contained both CD90.2^–^ and CD90.2^+^ subpopulations, whereas post-sort analysis demonstrated enrichment of the targeted fractions with minimal carry-over of the opposite population. **(C)** Quantitative summary of flow cytometry data, including total event counts and gating hierarchy. Percentages represent the proportion of CD90.2^–^ and CD90.2^+^ events within the live singlet gate.

1. Wash the cells with PBS and detach with Trypsin-EDTA (3 min, 37 °C).
2. Monitor cell detachment under the microscope and extend or repeat the incubation if necessary.
3. Quench enzymatic activity by adding two volumes of standard medium to the cell suspension.
4. Centrifuge at 200 *g* for 5 min at RT and discard the supernatant.
5. Resuspend the pellet in 1 ml ice-cold FACS buffer and count the number of cells.
6. Centrifuge again at 200 *g* for 5 min at RT and discard supernatant.
7. Resuspend the pellet in an ice-cold FACS buffer at a concentration of 10^6^ cells per 100 μl.
8. Incubate the cells with FITC-conjugated anti-mouse CD90.2 (Thy-1.2) antibody (Biolegend, 140303; 1 μl antibody per 1 * 10^6^ cells) on ice, in the dark for 20 min.
9. After incubation, add 5 ml of ice-cold FACS buffer and centrifuge at 200×*g* for 5 min at RT.
10. Resuspend the cells at 1 * 10^6^ cells per 500 μl of ice-cold FACS buffer, using a minimal volume of 250 μl.
11. Transfer the suspension through a cell-strainer cap into a sterile 5 ml polystyrene round-bottom FACS tube and keep on ice until sorting.
12. Carefully aspirate approximately two-thirds of the standard medium from the pre-coated polypropylene collection tubes and place them in the sort collection chamber.
13. Perform cell sorting using a BD FACSAria Fusion or an equivalent high-speed cell sorter equipped with a 100 μm nozzle.
14. Set gates using unstained controls, and instrument setup is performed according to the manufacturer’s recommendations using appropriate calibration beads.
15. Exclude debris and cell aggregates by sequential gating based on forward and side scatter (FSC-A vs. SSC-A), followed by singlet discrimination (e.g., FSC-H vs. FSC-A, Figures 2B,C).

1. Set a threshold FSC around 5,000 to minimize noise, perform sorting at a rate of approximately 1,500 events/s. To detect fluorescence in the FITC channel, use the 488 nm blue laser.
2. Use CD90.2 as a fibroblast-enriched marker to enable negative selection by removing CD90.2^+^ cells and collect the CD90.2^–^ fraction as the Schwann cell-enriched population.
3. Verify purity, this protocol typically achieves a Schwann cell purity > 97% (Table 2).

**TABLE 2:** Results of 4 independent sorting experiments (%Parent).

Experiment	Before FACS % Schwann cells	After FACS % Schwann cells
1	40	98.4
2	43	98.4
3	47.8	97.6
4	51.2	98.9

(E) Post-sorting procedures processing and cell seeding

For continued culture:

1. Centrifuge the collected cell fractions in the coated polypropylene tubes at 200 *g* for 5 min at RT.
2. Resuspend the pellet in 1 ml of fresh Schwann cell medium and count the cells using a Fuchs-Rosenthal chamber and trypan blue.
3. Seed the cells onto PLL-coated T25 flasks (at least 130,000 cells) or at a density of 5,000–15,000 cells per well for PLL-coated 24-well plate coverslips.

For direct analysis:

For applications such as qPCR or western blot, centrifuge the cell suspension to obtain a dry pellet or proceed directly to lysis, depending on the downstream workflow.

### 3.4 Characterization of Schwann cell and fibroblast populations

#### 3.4.1 Quantitative polymerase chain reaction

qPCR was performed to assess the enrichment of Schwann cell and fibroblast populations following FACS sorting. Sorted primary mouse cells (30 * 10^3^) were seeded on PLL-coated 24-well plates and allowed to adhere overnight in Schwann cell medium. Total RNA was extracted using QIAZOL Lysis reagent (Qiagen, Hilden, Germany) and the NZY RNA isolation kit (NZYTech, Lisboa, Portugal) according to the manufacturer’s instructions. RNA concentration and purity were determined with a Nanodrop spectrophotometer (Thermo Fisher Scientific). cDNA was synthesized from RNA at a final concentration of 3 ng/μl using Qscript reverse transcriptase (Quantabio, Beverly, MA, USA) using a T-100 Thermal Cycler (Bio-Rad Laboratories, Hercules, CA, USA). qPCR reactions were prepared using a SYBR Green master mix (Applied Biosystems, Thermo Fisher Scientific), and gene-specific primer pairs (Table 3). Ct values were determined using QuantStudio (Applied Biosystems, Thermo Fisher Scientific), and normalized against geNorm-validated. Each experiment included both CD90.2^–^ and CD90.2^+^ populations derived from pooled material from 2 mice.

**TABLE 3:** Mouse primer sequences for the qPCR analysis.

Gene	Forward primer sequence 5′-3′	Reverse primer sequence 5′-3′
Mpz	TCTCAGGTCACGCTCTATGTC	GCCAGCAGTACCGAATCAG
P75	CCCTGCCTGGACAGTGTTAC	ACAGGGAGCGGACATACTCT
Cd90	ATCCAAGTCGGAACTCTTGGC	GGACACCTGCAAGACTGAGAG
Pdgfrα	GCAGTTGCCTTACGACTCCAGA	GGTTTGAGCATCTTCACAGCCAC
Gapdh	ACCACAGTCCATGCCATCAC	TCCACCACCCTGTTGCTGTA

Mpz: myelin protein zero, P75: neurotrophin receptor for nerve growth factor, Cd90: cluster of differentiation 90, Pdgfrα: platelet-derived growth factor receptor alpha, Gapdh: glyceraldehyde-3-phosphate dehydrogenase.

#### 3.4.2 Immunocytochemistry

For immunocytochemical analyses, sorted cells were seeded at 10–15 * 10^3^ cells per well on PLL-coated glass coverslips in a 24-well plate, and cultured for 48 h (1/10 PLL, Sigma-Aldrich). Cells were washed with PBS and fixed with 4% paraformaldehyde for 20 min. Following fixation, cells were permeabilized with 0.05% Tween-20 in PBS for 15 min and subsequently blocked with 10% protein block serum-free (Dako, Agilent Technologies, Glostrup, Denmark) for 1 h at RT. Cells were incubated overnight at 4 °C with primary antibodies against P75, CD90.2, and PDGFRα (Table 4) in a humidified chamber. After repeated PBS washes, cells were incubated with the appropriate secondary antibodies (1/400; 1 h at RT). Negative controls lacking the primary antibody were included in each experiment. Nuclei were counterstained with Hoechst (Immunochemistry Technologies, CA, USA) for 20 min at RT, and coverslips were mounted using Immunomount (Thermo Fisher Scientific). Fluorescence images were captured using a Nikon Eclipse Ti2-E inverted microscope (Nikon Instruments) equipped with a CFI Plan-ApoChromat Lambda 20×/0.75 objective and a Photometrics Kinetix sCMOS camera, controlled by NIS-Elements Software. Signal intensity was quantified using ImageJ (Fiji, Version 2.14.0/1.54p) by measuring the integrated density, which was then normalized to cell number. To ensure a comprehensive analysis, the entire coverslip was imaged for each sample, with total cell counts per coverslip ranging from 400 to 20,000. For visualization purposes, brightness and contrast were adjusted equally across corresponding images within each staining condition, without altering the underlying quantitative analysis. To account for inter-experimental variability, data normalization was performed independently for each experiment to the mixed (unsorted) population. Each experiment included both CD90.2^–^ and CD90.2^+^ populations derived from pooled material from 10 mice.

**TABLE 4:** Antibodies used for western blot analysis, flow cytometry, or immunocytochemistry.

Protein	Application and dilution	Company (reference)
P75	ICC: 1/250 WB: 1/1,000	Cell signaling technologies (8238)
CD90.2	ICC: 1/250	Invitrogen (MA5-16683)
CD90.2-FITC	FACS: 1/100 WB: 1/1,000	Biolegend (140303)
PDGFRα	ICC: 1/250 WB: 1/500	Santa-cruz (sc398206)Abcam (ab134123)
MPZ	WB: 1/1,000	Abcam (ab183868)
β-actin	WB: 1/1,000	Novus biologicals (NB600-503SS)

P75: neurotrophin receptor for nerve growth factor, CD90.2: cluster of differentiation 90.2, PDGFRα: platelet-derived growth factor receptor alpha, MPZ: myelin protein zero.

#### 3.4.3 Western blot

For protein isolation, cells were incubated with RIPA buffer supplemented with a protease inhibitor cocktail (1/10 Roche Diagnostics, Indianapolis, IN) for 20 min on ice. Next, lysates were centrifuged at 13,000 *g* for 10 min at 4 °C and supernatants were stored at −80 °C until analysis. Total protein concentrations were determined using the Pierce BCA assay (Thermo Fisher Scientific) and absorbance was measured at 562 nm with an iMark Microplate Reader (Bio-Rad Laboratories). 5 μg of protein per sample and Precision Plus Protein Dual Color Standard ladder (Bio-Rad Laboratories) were loaded on 12% SDS-PAGE gels and separated at 100–200 V for 30–60 min. Proteins were transferred to polyvinylidene difluoride (PVDF) membranes at 350 mA for 1.5 h. Membranes were blocked in 5% non-fat milk (Marvel, Premier Foods, Dublin, Ireland) or 3% Bovine Serum Albumin (BSA, Biowest) in PBS for 1 h at RT and incubated overnight at 4 °C with primary antibodies against myelin protein zero (MPZ), P75, platelet-derived growth factor receptor alpha (PDGFRα), and CD90.2 (Table 4). The following day, blots were washed with 0.05% Tween-20 in PBS and incubated with horseradish peroxidase (HRP)-conjugated secondary antibodies (1/2,000) in 5% Marvel or 3% BSA in PBS for 1 h at RT. Detection was performed using the ECLPlus SuperSignal™ West Atto Ultimate Sensitivity Substrate or Pierce™ ECL Western Blotting (Thermo Fisher Scientific) on an Amersham Imager 680 (Cytiva, Marlborough, MA, USA). After detection, membranes were stripped and again incubated with β-actin as a loading control. Band intensities were quantified with ImageQuant TL 8.2 software (Cytiva) and normalized to β-actin. Each experiment included both CD90.2^–^ and CD90.2^+^ populations derived from pooled material from 10 mice.

#### 3.4.4 Statistical analysis

Statistical analyses were performed using GraphPad Prism version 9.3.1 (GraphPad Software, San Diego, CA, USA). Outliers were identified using the Grubbs’ test, and data normality was assessed using the Shapiro–Wilk test. For ICC data, statistical comparisons were conducted using the Kruskal–Wallis test (non-parametric one-way ANOVA). For qPCR and western blot analyses, statistical comparisons were performed using multiple Mann–Whitney tests, with correction for multiple comparisons using the Holm–Šídák method. Data are presented as mean ± SEM, and differences were considered statistically significant when *p* ≤ 0.05.

## 4 Results

### 4.1 Schwann cell morphology and purity before and after sorting

Mixed cultures obtained after short-term recovery displayed heterogeneous morphology, including elongated bipolar cells and larger polygonal cells. Following CD90.2-based sorting, the two fractions showed clearly distinct appearances (Figure 2A). The CD90.2^–^ fraction predominantly consisted of slender, spindle-shaped cells with bipolar or tripolar extensions, consistent with Schwann cell morphology, whereas the CD90.2^+^ fraction was mainly composed of large, flattened polygonal cells typical for fibroblasts (Figure 2A). Flow cytometry confirmed a clear separation of the two populations based on CD90.2 expression (Figure 2B). Prior to sorting, both CD90.2^–^ and CD90.2^+^ cells were present in the cultures (43.3% and 49.3%, respectively), whereas post-sort analysis demonstrated enrichment of the targeted fractions with minimal carry-over of the opposite population (Figure 2C). Purity across independent experiments is summarized in Table 2.

### 4.2 Validation of cell identity by gene expression analysis

**FIGURE 3:**
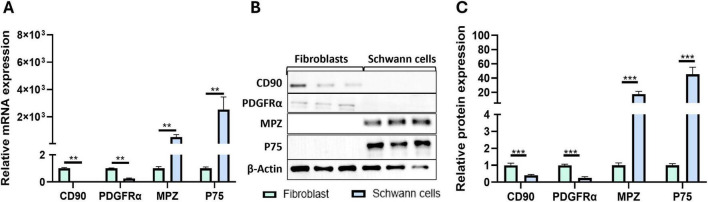
Validation of fibroblast and Schwann cell populations following sorting. **(A)** qPCR analysis of fibroblast-associated markers (*Cd90*, *Pdgfrα*) and Schwann cell markers (*Mpz*, *P75*) in CD90.2^+^ and CD90.2^–^ fractions. Data represent *n* = 3 independent experiments, using pooled material from 2 mice per experiment. **(B,C)** Representative western blot and quantification showing expression of Schwann cell markers (MPZ and P75) predominantly in the CD90.2^–^ fraction and fibroblast markers (CD90.2, PDGFRα) in the CD90.2^+^ fraction. β-Actin was used as a loading control. Western blot data were obtained from 3 to 4 independent experiments, using pooled material from ten mice per experiment. Statistical comparisons were performed using multiple Mann–Whitney tests, with appropriate correction for multiple comparisons using the Holm–Šídák method. Data are presented as fold change ± SEM, relative to the CD90.2^+^ (fibroblast) population (**p < 0.01, ***p < 0.001).

To evaluate lineage identity at the transcript level, expression of Schwann cell-associated markers (Mpz, P75) and fibroblast-associated markers (Cd90, Pdgfrα) was assessed by qPCR in both fractions (Figure 3A). The CD90.2^–^ population showed higher expression of Schwann cell markers (Mpz, P75), whereas the CD90.2^+^ population displayed increased levels of fibroblast-associated transcripts (Cd90, Pdgfrα). These differences were consistently observed across independent biological replicates.

### 4.3 Protein-level confirmation by western blot

Western blot analysis was performed to further examine marker expression in the sorted populations (Figures 3B,C). Lysates from the CD90.2^–^ fraction showed significantly higher levels of canonical Schwann cell marker proteins (MPZ, P75), while fibroblast-associated proteins were predominantly observed in the CD90.2^+^ fraction (CD90.2, PDGFRα). β-Actin was used as a loading control. These data provided protein-level validation of the segregation observed at the mRNA level.

### 4.4 Single-cell validation by immunocytochemistry

**FIGURE 4:**
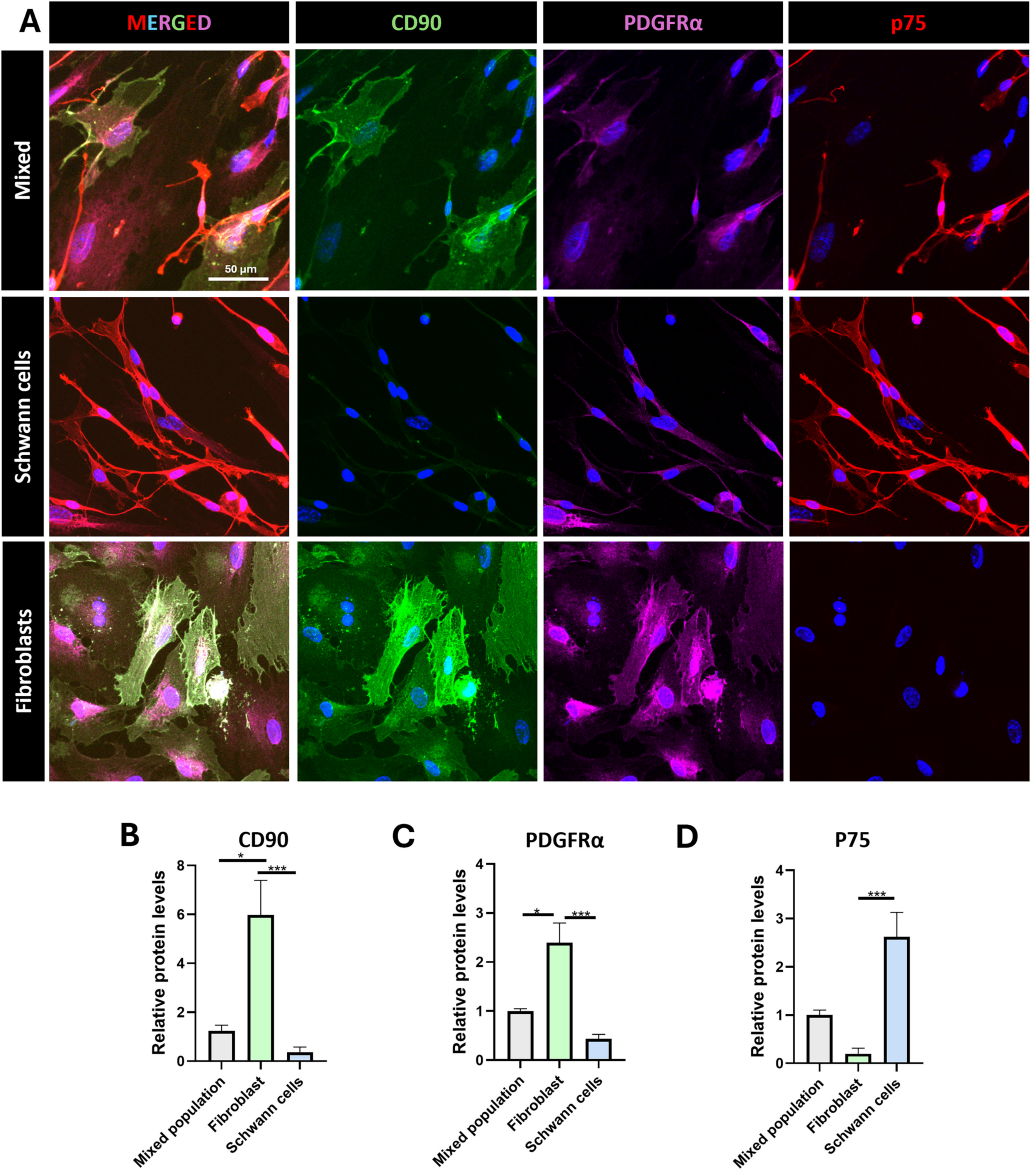
Immunocytochemical validation of CD90.2-guided sorted populations. **(A)** Representative fluorescence images of mixed cultures prior to sorting (upper panel), the CD90.2^–^ Schwann cell-enriched fraction (middle panel), and the CD90.2^+^ fibroblast-enriched fraction (lower panel). Schwann cells display an elongated bipolar morphology and P75 positivity with limited CD90.2/PDGFRα signal, whereas fibroblasts show the opposite staining pattern. Nuclei are counterstained with Hoechst. Scale bar = 50 μm. **(B–D)** Quantification of integrated fluorescence intensity for CD90.2 **(B)**, PDGFRα **(C)**, and P75 **(D)**. Data are derived from 3 independent experiments, using pooled material from ten mice. Data were analyzed using a Kruskal–Wallis test (non-parametric one-way ANOVA). Data are presented as fold change ± SEM, relative to the mixed population (*p < 0.05, ***p < 0.001).

Immunocytochemical staining was used to visualize marker distribution at single-cell resolution (Figure 4). Mixed cultures displayed heterogeneous marker expression, with cells positive for both Schwann cell and fibroblast-associated markers. The CD90.2^–^ fraction contained predominantly P75-positive cells with elongated morphology, whereas the CD90.2^+^ fraction was largely positive for CD90.2 and PDGFRα. Although PDGFRα staining was markedly stronger in the CD90.2^+^ fraction, low-level PDGFRα signal could also be detected in the CD90.2^–^ fraction. Quantification of integrated fluorescence intensity confirmed significantly different expression levels of these markers between the two fractions. Negative controls lacking primary antibody showed no specific signal.

## 5 Troubleshooting

To ensure maximum yield and purity, we have outlined key troubleshooting steps for the enrichment of Schwann cells in Table 5, addressing common pitfalls in the procedural workflow.

**TABLE 5:** Troubleshooting.

Problem	Action and rationale
Viability and yield	
Mechanical and enzymatic stress	Prioritize enzymatic digestion over extensive teasing as it is less detrimental to membrane integrity for adult nerve tissue. Optimizing enzymatic digestion conditions (e.g., reducing digestion time, using alternative enzymes such as general collagenase and neutral protease) can improve yield compared to purely mechanical approaches. Additionally, monitor trypsinization via phase-contrast microscopy and stop immediately once cells round up to prevent over-digestion.
pH instability	Increase CO_2_ to a 8–9% atmosphere for bicarbonate-buffered media (DMEM) to prevent alkalinization. Use phenol red as a visual indicator to monitor pH stability (Andersen and Monje, 2018).
Cell loss during isolation	Use glass or FBS/BSA-coated plasticware. Schwann cells easily adhere to standard plastic, leading to significant loss during enzymatic steps and transfers (Castaneda et al., 2023).
Labile growth factors	Use medium < 48 h old. Growth factors like bFGF known to be highly thermolabile and lose mitogenic potency rapidly when stored in diluted form. Using fresh medium ensures a robust mitogenic stimulus (Gospodarowicz and Cheng, 1986; Benington et al., 2020, 2021).
Scaling	Pool nerves from at least two mice. FACS requires a minimum tissue quantity to reliably identify and gate the target population.
Low attachment	
Poor seeding success	Alter droplet plating by seeding cells in small droplets to increase effective seeding density. This promotes rapid attachment and facilitates separation from floating myelin debris (Andersen and Monje, 2018; Aparicio and Monje, 2023).
Suboptimal surface	If using PLL, wash thoroughly with sterile MQ, residual PLL is cytotoxic at high concentrations (Zhu et al., 2023). Alternatively, switch to laminin coating for superior adhesion (Niapour et al., 2010; Andersen and Monje, 2018; Aparicio and Monje, 2023).
Low purity and sorting issues	
Myelin and debris contamination	A short culture period (7–10 days) prior to sorting allows for cellular clearance of myelin (see Figures 1D–E) and prevents fibroblast overgrowth (Aparicio and Monje, 2023). Increase medium changes to 5×/week to wash away extracellular debris.
Sorter clogging	Filter the cell suspension through 35–40 μm strainers prior to sorting to remove aggregates, and use a 100 μm nozzle to minimize shear stress and clogging. Dilute the cell suspension in larger FACS buffer volumes to minimize nozzle obstruction.
Poor SC/fibroblast separation	Titrate antibodies and include unstained controls to define gating boundaries (Basu et al., 2010). Use viability dyes (e.g., 7-AAD) to exclude dead cells, which can bind non-specifically (Cossarizza et al., 2021). Add PDGFRα (CD140a) as an extra exclusion marker for fibroblasts.
Doublet interference	Apply rigorous doublet discrimination (FSC-H vs. FSC-A) to prevent co-sorting of Schwann cells adhered to fibroblasts.

## 6 Discussion

Primary Schwann cell cultures remain an indispensable model for studying peripheral nerve biology, nerve repair, and myelination (Ten Asbroek et al., 2006; Endo et al., 2022; Negro et al., 2022). However, fibroblast overgrowth remains a persistent bottleneck that compromises both reproducibility and purity (Manent et al., 2003; Monje, 2023). This method complements existing approaches by prioritizing cellular integrity over maximal yield or throughput (Andersen et al., 2016). Traditional enrichment strategies often rely on differential adhesion or on antimitotic agents to selectively eliminate proliferating fibroblasts. These methods are frequently suboptimal, as they require extended culture periods, risk significant Schwann cell toxicity, and can irreversibly alter the metabolic state of the surviving glia (Brockes et al., 1979; Calderón-Martıìnez et al., 2002; Kisselbach et al., 2009). Alternative biological selection techniques have been developed, but each presents specific limitations.

MACS-based positive selection, for instance, necessitates direct receptor ligation of the target Schwann cell population, which may induce receptor clustering and downstream signaling, potentially altering the cellular state (Vroemen and Weidner, 2003; Elkord et al., 2005; Andersen et al., 2016). Similarly, immunopanning, while often regarded as the gold standard for high-purity glial cultures, presents significant logistical and physiological challenges (Lutz, 2014; He et al., 2020; Nolle et al., 2021). Although effective for sequential cell selection, it is a low-throughput and technically demanding process. Prolonged incubation steps can result in cell loss and cellular stress. In addition, extended antibody-receptor interactions (e.g., P75) may alter the basal physiological state of Schwann cells and influence downstream functional assays (Elkord et al., 2005; Vilar et al., 2009; Beliakova-Bethell et al., 2014). Such receptor engagement may trigger intracellular signaling responses, which could confound assays aimed at assessing the baseline functional state of Schwann cells (Cragnolini and Friedman, 2008; Vilar et al., 2009; Beliakova-Bethell et al., 2014). Our approach, utilizing a CD90.2-based negative selection strategy via FACS, addresses these shortcomings by focusing on the depletion of fibroblast contamination. A key methodological benefit of this strategy is the targeting of the fibroblast lineage through CD90.2 (Thy-1.2), thereby avoiding Schwann cell surface marker ligation and lengthy adhesion steps. This supports preservation of a more native cellular profile, however, whether it better preserves the Schwann cell transcriptomic profile compared to positive selection approaches was not directly assessed in the present study and remains to be evaluated by dedicated transcriptomic analyses.

In this regard, MACS-based negative selection would be a possible technique to isolate primary Schwann cells, as it avoids direct target ligation. However, while each cell isolation technique offers distinct advantages depending on the experimental scale and required throughput, FACS offers superior precision over MACS and immunopanning through the possibility of multi-parametric gating (Xu et al., 2014; Sutermaster and Darling, 2019; Pan and Wan, 2020). Hence, FACS further enables efficient depletion of fibroblast subpopulations that may escape binary magnetic separation (Basu et al., 2010; Staunstrup et al., 2022). The incorporation of viability dyes enables the specific exclusion of dead cells and debris, thereby ensuring a healthier starting population. Moreover, by avoiding direct antibody binding to Schwann cell surface markers, the risk of selection-induced artifacts is reduced. Importantly, surface epitopes remain available for downstream applications such as immunostaining or ligand-binding assays (Elkord et al., 2005; Vilar et al., 2009; Basu et al., 2010; Beliakova-Bethell et al., 2014). This approach consistently yields highly enriched Schwann cell populations, even from anatomically complex tissues such as the brachial plexus.

However, these advantages must be weighed against several technical drawbacks. The required 7–10 day pre-sorting culture introduces a risk of phenotypic drift, limiting applicability for acute transcriptomic analyses (Tomlinson et al., 2020). The use of Schwann cell medium supplemented with growth factors for this recovery phase was based on established adult Schwann cell culture protocols designed to support cell survival and growth rate after nerve dissociation (Andersen et al., 2016; Andersen and Monje, 2018; Aparicio and Monje, 2023). While the specific impact of these culture conditions on Schwann cell transcriptional profiles was not assessed in the present study, previous studies using comparable adult Schwann cell culture conditions reported relatively homogeneous expression of Schwann cell-specific markers and preservation of characteristic Schwann cell properties, including neuregulin-dependent proliferation and the ability to induce myelin-specific gene expression upon stimulation (Andersen et al., 2016). In addition, shear stress during sorting may affect cell viability and phenotype (Pfister et al., 2020). In our hands, varying sorting pressures and nozzle sizes did not markedly affect viability, suggesting relative resilience to sorting conditions. However, Schwann cells may still be more sensitive to mechanical stress than more robust cell types (Gupta et al., 2005). Access to specialized instrumentation and expertise may limit broader implementation. In the present protocol, PLL was used as a simple and broadly accessible baseline coating. However, laminin, either alone or in combination with PLL, may further improve Schwann cell adhesion and spreading and should be considered as an optimization step for applications requiring enhanced post-sort attachment (Niapour et al., 2010; Andersen et al., 2016; Andersen and Monje, 2018; Aparicio and Monje, 2023). Importantly, the CD90.2^–^ fraction is not exclusively composed of Schwann cells. Residual non-glial cells from the endoneurial niche may be co-isolated. Single-cell transcriptomic analysis of the peripheral nerve indicates that while Schwann cells and fibroblasts constitute the majority of the population (∼65%), vascular (CD31^+^, ∼20%) and hematopoietic/immune cells (CD45^+^, ∼15%) are also natively present (Wolbert et al., 2020; Yim et al., 2022). Specifically, microvascular capillaries (CD31^+^) and a population of interstitial resident macrophages (CD45^+^) may be co-isolated (Ren et al., 2024). However, these minor populations are typically lost during early culture due to the lack of cell-specific growth factors and the selective nature of the Schwann cell medium (Yu et al., 2012). This may also explain the presence of a small subset of cells lacking detectable expression of either Schwann cell or fibroblast-associated markers in the immunocytochemistry analyses. Nevertheless, a distinct advantage of our FACS-based strategy is its inherent flexibility, adding additional markers such as CD31 and CD45 could easily be integrated to refine isolation. Finally, protein analyses were performed on pooled samples, limiting assessment of inter-individual variability. In addition, an assessment of post-isolation viability and proliferation was not included in this study and could further facilitate comparison with alternative isolation protocols. Future studies could evaluate whether alternative CD90.2 antibody clones perform similarly in this workflow.

In conclusion, the optimized CD90.2-based negative selection protocol provides a robust method for obtaining high-purity primary mature murine Schwann cells while prioritizing cellular integrity and a naïve physiological state. Consequently, this protocol is particularly suited for applications requiring high molecular sensitivity and untouched surface receptors, such as ligand-binding assays, cell-based drug screening, co-culture myelination studies and other functional assays. While residual fibroblast proliferation remains a consideration in long-term cultures, this approach provides a robust and reproducible platform for advanced peripheral nerve research.

## Data Availability

The raw data supporting the conclusions of this article will be made available by the authors upon reasonable request.
